# Effect of Aerobic Exercise with Blood Flow Restriction on Postexercise Hypotension in Young Adults: The Role of Histamine Receptors

**DOI:** 10.3390/jcdd11100326

**Published:** 2024-10-15

**Authors:** Dongnyeuck Seo, Dae Sik Song, William Boyer, Trevor Gillum, Sean Sullivan, Nailiyah Liwanag, Iltark Yoon, Jong-Kyung Kim

**Affiliations:** Department of Kinesiology, California Baptist University, 8432 Magnolia Avenue, Riverside, CA 92504, USA; dongnyeuck.seo@calbaptist.edu (D.S.); daesik.song@calbaptist.edu (D.S.S.); wboyer@calbaptist.edu (W.B.); tgillum@calbaptist.edu (T.G.); ssullivan@calbaptist.edu (S.S.); nailiyahi.liwanag@calbaptist.edu (N.L.); iltark.yoon@calbaptist.edu (I.Y.)

**Keywords:** blood flow restriction, mean arterial pressure, histamine receptor, total peripheral resistance, cardiac output

## Abstract

We tested hypothesis that aerobic exercise with blood flow restriction (BFR) induced postexercise hypotension (PEH), and the reduction in blood pressure (BP) was due to peripheral vasodilation via the histamine receptors. Ten male subjects participated in this study. The subjects were randomly assigned to walk for 10 min at 6.4 km/h, 0% grade with or without BFR after taking histamine receptor blockade. Following exercise, BP was measured at 10 min interval for 60 min. Heart rate (HR), stroke volume (SV), cardiac output (CO), mean arterial pressure (MAP), and total peripheral resistance (TPR) were evaluated. Our results indicated that MAP was significantly lowered immediately after exercise at 20 min, 30 min, and 40 min before the blockade as opposed to after the blockade. A significant reduction in diastolic BP (DBP) occurred. There were no significant differences in HR, SV, CO, and TPR between before the blockade and after the blockade. MAP was substantially decreased at 20 min, 30 min, and 40 min before the blockade compared to resting (−3.2 ± 2.2, −3.3 ± 2.8, and −2.9 ± 2.5, respectively) while increasing MAP after the blockade. The current study demonstrated that low-intensity aerobic exercise with BFR lowered MAP via histamine receptor-induced peripheral vasodilation. In conclusion, BFR exercise training using short periods and low intensity would be greatly beneficial as a potential treatment to lower BP.

## 1. Introduction

High BP is a modifiable risk factor for developing cardiovascular disease [1]. Over the past decade, regular exercise has been known to be a potential approach for managing BP as a non-pharmacological treatment. It has been demonstrated through evidence in individuals and animals with normotension and hypertension that aerobic exercise training attenuated BP [2,3,4]. Specifically, a single session of light to moderate exercise induces PEH and this effect can persist for up to 24 h in humans [5]. PEH has been demonstrated following a variety of aerobic exercises such as walking, running, and cycling [6,7,8]. Together, long-term exercise training may benefit normotensive and hypertensive individuals by lowering BP for a longer period.

Because BP is the product of CO and TPR, the underlying mechanism that causes PEH has been attributed to either a reduction in CO or peripheral vasodilation [5]. Although systemic vasodilation contributes to PEH, importantly, the specific role of each receptor system still needs to be determined.

BFR exercise training was first introduced in Japan and is now widely practiced worldwide [9]. It involves a short-term low-intensity exercise with a tourniquet wrapped around the working muscles to perform occlusion [10]. BFR training is primarily performed in the setting of resistance exercise. It is well established that BFR training has similar benefits of high-intensity resistance exercise compared to exercising in low-intensity resistance. This maneuver substantially accumulates metabolites such as lactate and hydrogen ions within exercising muscles that improve muscle force, muscle growth, functional capacity, and muscular strength [11,12,13,14].

Studies demonstrated that BP was reduced following resistance exercise with BFR [15,16,17]. Most studies used a high volume of aerobic exercise lasting between 20 and 60 min at moderate or high exercise intensity. However, there are safety concerns because BFR training evokes markedly exaggerated increases in BP and limits the safety of exercise training [18]. Thus, an appropriate mode of training that can be used as a suitable alternative to high-intensity exercise has yet to be determined. Accordingly, low-intensity aerobic exercise with BFR can be a good maneuver to reduce BP since it is relatively safe.

It is well documented that histamine is released by mast cell degranulation and increased production from histidine decarboxylase (HDC) in response to elevated blood flow in reactive hyperemia, hypoxia, increased temperature, and a low pH induced by exercise [19,20,21]. Histamine H1 and H2 receptors contribute to PEH via postexercise vasodilation [22]. In agreement with this finding, a previous study has demonstrated that one hour of moderate-intensity exercise elevated histamine concentration within active muscle and elicited postexercise hyperemia via stimulation of peripheral H1 and H2 receptors in sedentary and endurance-trained individuals [23,24]. However, it is not known whether aerobic exercise with BFR can elicit PEH via histamine H1 and H2 receptors. Since it has been reported that a hypoxic condition increases the release of histamine [25] and enhances peripheral vasodilation, this study hypothesized that aerobic exercise with BFR induces PEH, and the reduction in BP following aerobic exercise with BFR was due to peripheral vasodilation via the histamine H1 and H2 receptors.

## 2. Materials and Methods

A total of 10 male subjects participated in this study. Only male subjects participated in this study to mitigate the potential influences of the menstrual cycle on exercise hemodynamics [26]. The subjects were recruited from the California Baptist University campus and the surrounding Riverside community. Subjects participating in this study were in good health, nonsmokers, and those not taking medications that could affect cardiovascular function. The subjects were instructed to abstain from alcohol, caffeine, and strenuous exercise for 24 h before every visit. All subjects signed an informed consent form and were screened for cardiovascular disease risk factors through a health history questionnaire (HHQ). All procedures and protocols were reviewed and approved by the California Baptist University Institutional Review Board (141-2324-EXP; May 2024). This study was registered in Clinicaltrials.gov (NCT06629337).

### 2.1. Experimental Procedures

This study is designed as a randomized cross-over trial. Subjects reported to the laboratory a total of 3 times. On the first visit for screening, the subjects wrote an informed consent form and an HHQ to screen their cardiovascular disease risk factors. Then, their BP and anthropometric data (height and weight) were measured. The subjects rested quietly for 30 min, and BP was measured in the sitting position. Three BP measurements (using an appropriately sized pressure cuff) are obtained every 5 min using a mercury sphygmomanometer with the cuff positioned on the left arm at the level of the heart. At the same time, the subjects sat with their backs supported on the chair and both feet on the floor. Following the screening, the subjects were randomly assigned to two aerobic exercise trials: first, aerobic exercise using BFR without histamine receptor blockade; second, aerobic exercise using BFR with histamine receptor blockade. The trials were conducted on two separate days, 48–72 h apart.

### 2.2. Exercise Protocol

Every subject performed a 10 min aerobic exercise on the treadmill with a speed of 4 mph and 0% grade [27]. Ratings of perceived exertion (RPE) was asked throughout the exercise every 1 min. The subject’s BP was measured immediately following the exercise, and they were instructed to sit on a chair for an hour with electrodes attached. Their BP was measured every 10 min, and their hemodynamic response was screened throughout the process through non-invasive measurement using impedance cardiography (Physio Flow, Petit Ebersviller, France). This process was applied to all trials with BFR tourniquets applied (BFR Therapy, North Las Vegas, NV, USA). The tourniquet was placed on both upper thighs and inflated to a pressure of 140 mmHg and deflated immediately after exercise. Lastly, the subjects took two histamine receptor-blocking pills.

Histamine H1 receptor blockade was induced by fexofenadine 540 mg (Allegra; Pfizer Consumer Healthcare, Morris Plains, NJ, USA), and H2 receptor blockade was induced by famotidine 40 mg (Pepcid AC, Parsippany, NJ, USA). Subjects ingested fexofenadine at least 50 min before and famotidine 1 h 50 min before the onset of each protocol because these doses of oral fexofenadine and famotidine reach their peak concentrations at around 1 h and 2 h, respectively [28,29,30]. Administering these amounts of histamine receptor blockades leads to the inhibition of over 90% of histamine H1 and H2 receptors for 6 h [29]. The washout period between the absence and presence of histamine blockades of each trial was separated by at least 2 days because fexofenadine takes 2–3 days and famotidine takes less than 24 h to be completely removed from the body.

### 2.3. Measurement of Hemodynamic Responses

Non-invasive measurements of SV, HR, and CO were acquired using impedance cardiography (Physio Flow, Manatec Biomedical, Paris, France) before and after intervention. This technique evaluates alterations in thoracic impedance to derive hemodynamic responses [31]. In brief, the process entails the transmission of a high-frequency, low-magnitude alternating current (AC) through two sets of skin electrodes. MAP was calculated from SBP and DBP using the formula MAP = [(SBP − DBP)/3] + DBP. TPR was calculated as MAP/CO. All BP measurements in each subject were measured by the same investigator.

### 2.4. Statistical Analysis

Changes in all variables were expressed as means ± SD. The normality of the sample was performed using a Shapiro–Wilk test. The test revealed that all measured variables were normally distributed. The one-minute average values of HR, SV, and CO were collected before and after the blockade at rest and every 10 min following BFR exercise for 60 min. SBP and DBP were measured at rest, immediately after exercise, and every 10 min. For hemodynamic data obtained at rest and after exercise, two-way repeated-measures ANOVA was used for comparison with respect to the blockade and time. If significant interaction was found, a test for a Tukey’s post hoc analysis was performed to determine significant group mean differences. A one-way repeated-measures ANOVA (SIGMASTAT 4.0, Tulsa, OK, USA) was used for comparison with respect to the resting BP and PEH. If significant interaction was found, a test for a Tukey’s post hoc analysis was performed to determine significant mean differences over time. A sample size calculation (power = 0.80; α = 0.05) using G*Power 3.1 software indicated that 10 subjects were needed to obtain a difference of 5 mmHg in SBP after BFR exercise [32]. Statistical significance was accepted at *p* < 0.05.

## 3. Results

Table 1 represents the physical characteristics of subjects. All subjects had normal BP at rest.

Table 2 showed HR, SV, CO, SBP, DBP, MAP, and TPR at rest before and after the blockade. There were no significant differences in physiological responses between the two conditions.

**Figure 1:**
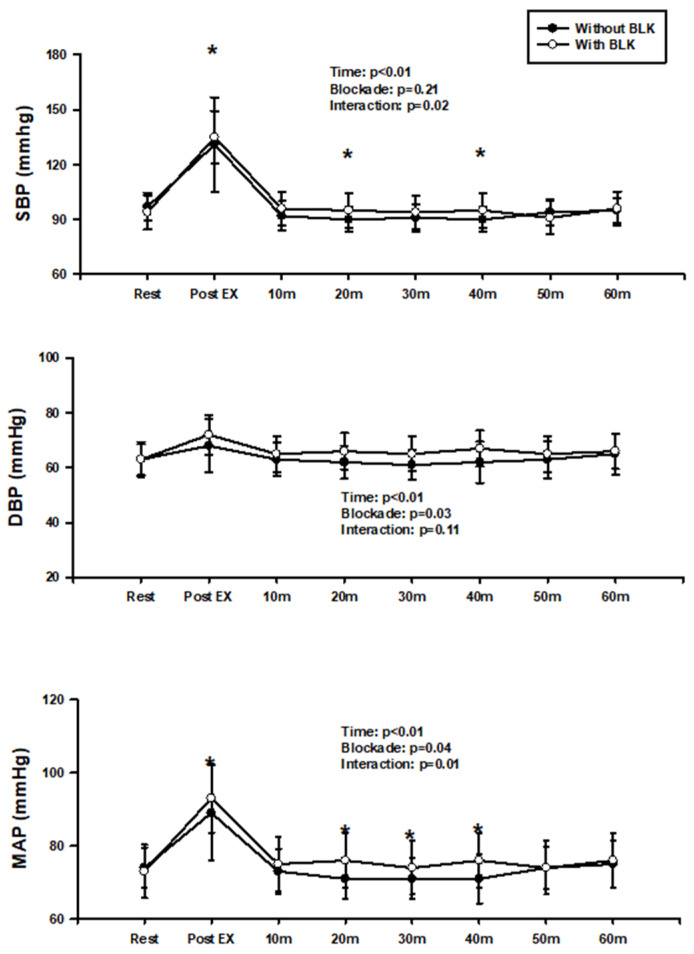
SBP, DBP, and MAP at rest and after BFR exercise before and after the blockade. * *p* < 0.05, vs. blockade.

Figure 1 showed the result of two-way repeated-measures ANOVA on SBP, DBP, and MAP at rest and following BFR exercise before and after blockade. There was a significant interactive effect in both SBP and MAP, implying that SBP was significantly lower at immediately after exercise, 20 min and 40 min before the blockade compared to after the blockade. MAP was significantly lower at immediately after exercise, 20 min, 30 min, and 40 min. Although no interaction was observed, significant time and blockade effects in DBP occurred.

**Figure 2:**
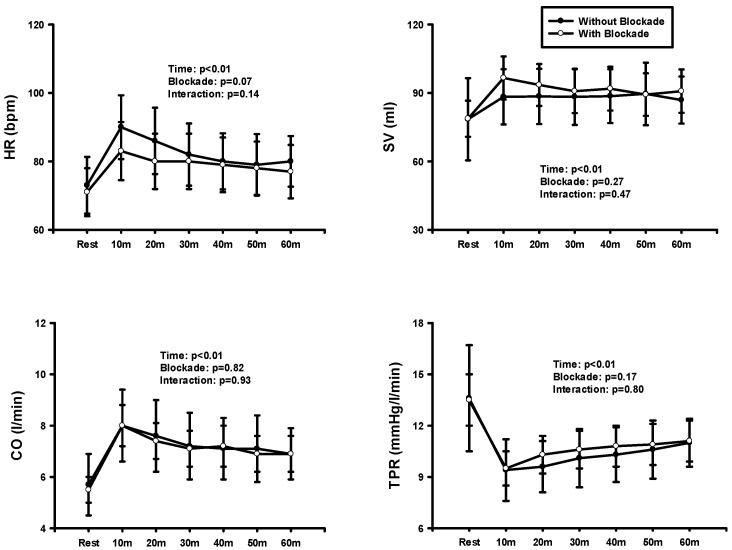
HR, SV, CO, and TPR at rest and after BFR exercise before and after the blockade.

Figure 2 showed the result of two-way repeated ANOVA on HR, SV, CO, and TPR at rest and after exercise before and after blockade. HR was significantly higher after the blockade. There was no significant interaction, but significant time and blockade effects in both HR and CO were observed. HR and CO significantly increased in both conditions over time, but these variables were significantly elevated after the blockade compared with before the blockade. Significant time effects occurred in both SV and TPR. These variables were significantly higher in SV over time compared to the rest while lower in TPR.

**Figure 3:**
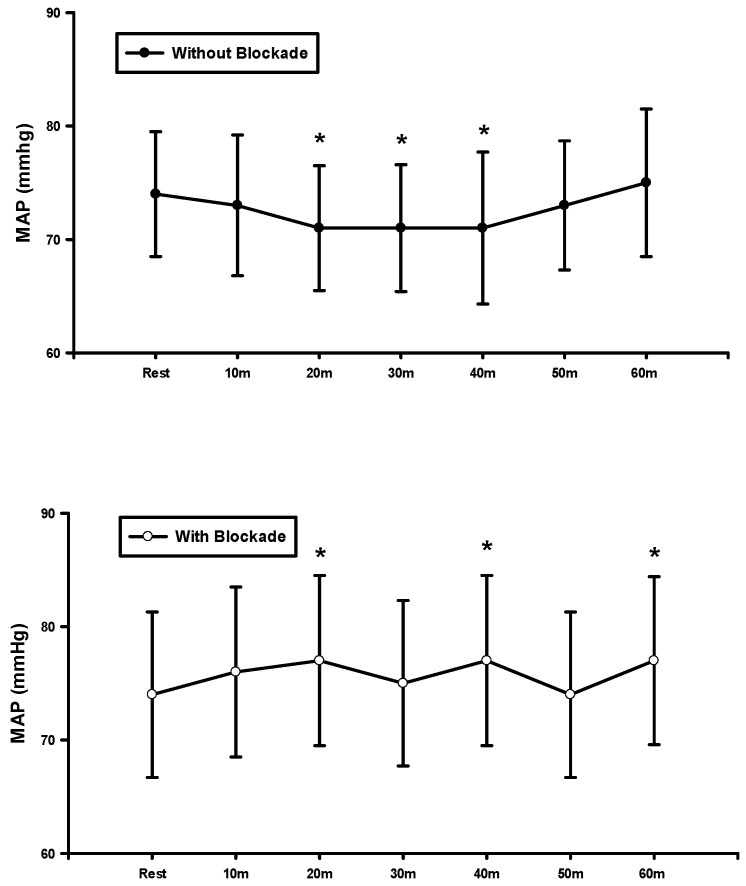
MAP at rest and after BFR exercise before and after the blockade. * *p* < 0.05, vs. rest.

Figure 3 showed the result of one-way repeated-measures ANOVA between the baseline and postexercise MAP before and after blockades. MAP was significantly lower at 20, 30, and 40 min without blockade, indicating that BFR exercise elicited PEH. On the other hand, MAP was significantly higher at 20 and 40 min after the blockade, implying that the histamine receptors contributed to a reduction in BP following the BFR exercise.

## 4. Discussion

We investigated the effects of aerobic exercise with BFR on PEH and the role of histamine receptors in mediating BP responses in normotensive individuals. The new findings of this study were that following BFR exercise, MAP was lower before the blockade compared to after the blockade. TPR was significantly decreased in two settings over time, but the TPR appeared to show more reduction before the blockade. CO was increased in two conditions after BFR exercise. Accordingly, the reduction in MAP was mainly due to increased peripheral vascular conductance. Furthermore, BP was substantially decreased following BFR exercise compared to the rest while increasing after the blockade. Thus, the histamine blockade blunted the reduction in BP. Collectively, the current study demonstrated that low-intensity and short-duration aerobic exercise with BFR induces PEH and that the histamine receptors play an important role in reducing BP.

The main purpose of this study was to quantify whether the reduction in arterial BP initiated by BFR exercise was primarily due to the contribution of CO or peripheral vasodilation. The results indicated that there was elevated CO concomitant with a reduction in TPR in both conditions over time. BFR exercise had a reduction in TPR, but there was no difference between the two conditions. Interestingly, a reduction in TPR concurrent with little increase in CO dropped MAP responses for ~40 min before the blockade. TPR is a calculated variable (MAP/CO). Theoretically, since the increased CO was offset by the reduced TPR, the MAP would remain similar before and after the blockade. Unexpectedly, MAP was lower before the blockade compared to after the blockade. It is logical to suggest that although there was no statistical difference in TPR between the two settings, reduced MAP was due to greater peripheral vasodilation before the blockade. It should be noted that BFR exercise caused partially peripheral vasodilation in exercising skeletal muscles, as demonstrated by the significant decrease in DBP in this study [33]. Taken together, the present study suggests that peripheral vasodilation plays a critical role in reducing BP responses mediated by aerobic exercise with BFR.

Although the precise mechanism of what causes PEH is still unknown, it has been suggested that there are some contributing factors such as reduced sympathetic output by the arterial baroreflex resetting [34] and local vasodilation mechanisms [22]. Of the local vasodilator substances, histamine receptors elicited PEH via postexercise vasodilation [35]. In support of this concept, a previous study demonstrated that PEH elicited by histamine H2 receptors mediated postexercise vasodilation after a single bout of dynamic exercise consisting of a 60 min period at moderate intensity, whereas the blockade blunted PEH [23]. In agreement with this finding, our results showed that acute BFR exercise resulted in sustained PEH responses (i.e., 20 min, 30 min, and 40 min). In contrast, the receptor blockade augmented MAP responses following BFR exercise. With the blockade, PEH is abolished, implying that the histamine receptors play a critical role in inducing PEH. Together, the major mechanism by which the aerobic exercise with BFR caused PEH is increasing peripheral vasodilation via histamine receptor activation.

This present study also found that HR, SV, and CO were higher following exercise in both conditions compared to pre-exercise. The mechanism by which these variables were elevated with PEH is still unclear. It has been reported that high temperature following exercise affects the sinoatrial node and enhances heart contractility [22].

### 4.1. Limitations of the Study

A limitation of this study is the fact that this study did not directly assess sympathetic output, which affects BP. It is established that exercise resets the arterial baroreflex to reduce sympathetic activity and lower BP. The specific effects of sympathetic output by the arterial baroreflex resetting BP need to be assessed. This study did not isolate only the contribution of histamine from other potential contributors to sustained PEH (i.e., prostaglandin and nitric oxide). However, previous studies reported that those substances had small effects on PEH [36,37]. Lastly, we did not measure BP responses during aerobic exercise with BFR. Some concerns are raised because BFR exercise evoked abnormal reflex-mediated cardiovascular responses. In this study, BP was measured immediately after exercise due to difficulty in measurement during exercise. SBP and DBP were 130 ± 8 and 68 ± 3 mmHg, respectively. It is important to consider this limitation when evaluating the safety of exercise training.

### 4.2. Perspective and Significance

This study suggests that low-intensity aerobic exercise with BFR can elicit a similar PEH effect in a much shorter time compared to other exercises that have been performed with moderate to high workloads lasting more than 20 min [23,24]. The efficiency of this protocol can be practical not only for normal healthy adults but also as a form of treatment and rehabilitation therapy in clinical settings. Previous studies reported a strong correlation between the magnitude of PEH and the long-term reductions in BP achieved through aerobic exercise training in prehypertensive individuals [38,39]. Thus, BFR exercise training using short periods and low intensity would be greatly beneficial as a potential treatment to lower BP and improve cardiovascular health.

## 5. Conclusions

The current study demonstrated that low-intensity aerobic exercise with BFR lowered MAP before the blockade compared to the blockade. The reduction in MAP was primarily due to peripheral vasodilation. This combination with histamine H1 and H2 receptors plays a pivotal role in sustained PEH following BFR exercise. Thus, walking with BFR can provide a potential therapeutic intervention for individuals suffering from high BP.

## Figures and Tables

**Table 1:** Physical characteristics of subjects.

Variables	(*n* = 10)
Age (yrs)	23.3 ± 2.8
Height (cm)	175.2 ± 6.5
Weight (kg)	72.2 ± 8.6
Body mass index (kg/m^2^)	23.6 ± 2.9
SBP (mmHg)	96 ± 6.5
DBP (mmHg)	63 ± 6.0
MAP (mmHg)	74 ± 5.5

Values are expressed as mean ± standard deviation.

**Table 2:** Hemodynamic data at rest before and after the blockade.

Values	(*n* = 10)	
	Control	Blockade
HR (bpm)	73 ± 8.3	71 ± 7.0
SV (mL)	79.8 ± 18.0	78.7 ± 7.9
CO (L/min)	5.8 ± 1.2	5.5 ± 0.5
SBP (mmHg)	98 ± 8	95 ± 9
DBP (mmHg)	64 ± 6	63 ± 6
MAP (mmHg)	75 ± 6	73 ± 7
TPR (mmHg/L/min)	13.6 ± 3.1	13.5 ± 1.5

Values are expressed as mean ± standard deviation.

## Data Availability

The data used in the analysis are available upon request to the corresponding author.
